# Multiday corticosteroids in cancer chemotherapy delay the diagnosis of and antimicrobial administration for febrile neutropenia: a double-center retrospective study

**DOI:** 10.1186/s40780-018-0130-2

**Published:** 2019-02-04

**Authors:** Hiroki Uda, Yukio Suga, Eriko Toriba, Angelina Yukiko Staub, Tsutomu Shimada, Yoshimichi Sai, Masami Kawahara, Ryo Matsusita

**Affiliations:** 1 Department of Clinical Drug Informatics, Faculty of Pharmacy, Institute of Medical, Pharmaceutical & Health Science, Kanazawa University, 13-1 Takaramachi, Kanazawa, Ishikawa 920-8641 Japan; 2 Department of Pharmacy, Kanazawa Municipal Hospital, 3-7-3 Heiwamachi, Kanazawa, Ishikawa 921-8105 Japan; 3 Department of Pharmacy, Kanazawa University Hospital, Kanazawa University, 13-1 Takaramachi, Kanazawa, Ishikawa 920-8641 Japan

**Keywords:** FN, Febrile neutropenia, Corticosteroids, Cancer chemotherapy, Body temperature

## Abstract

**Background:**

Medical staff should promptly administer antimicrobials to patients with febrile neutropenia (FN) to decrease the mortality related to cancer chemotherapy. Corticosteroids, which are used in cancer chemotherapy, have a fever-suppressive effect. This effect could lead to a blunt fever response and any local signs of infection, especially in patients receiving multiday corticosteroid administration. The aim of this study was to determine whether multiday corticosteroid administration in cancer chemotherapy delays the diagnosis of and antimicrobial treatment for FN.

**Methods:**

We conducted a double-center retrospective study in Japanese patients with FN. The patients were divided into two groups based on the corticosteroid administration method, i.e., whether administration was multiday or not. To evaluate the degree of masking on FN by corticosteroids, we assessed the correlation between body temperature variation and time of antimicrobial administration after the initiation of chemotherapy. Risk factors for delayed antimicrobial administration were identified by multiple logistic regression analysis.

**Results:**

Two hundred thirteen patients were analyzed. The median time required to body temperature reaching 37.5 °C and for antimicrobial administration was longer in the multiday group than in the non-multiday group, with 0.64 and 0.60 days (*P* = 0.002 and *P* < 0.001), respectively. Multiday corticosteroid use was identified as an independent risk factor for delayed antimicrobial administration (odds ratio = 3.94; 95% confidence interval = 1.80–8.62; *P* < 0.001).

**Conclusions:**

Multiday corticosteroid administration in cancer chemotherapy delayed the diagnosis of and antimicrobial administration for FN. Furthermore, it was the only risk factor for delayed antimicrobial administration. We could thus provide evidence that the diagnosis of and antimicrobial administration for FN in patients receiving multiday corticosteroid administration should not be based on body temperature variation alone.

## Background

Febrile neutropenia (FN) is the most serious adverse effect of cancer chemotherapy. This life-threatening complication results in dose reduction and delay of cancer chemotherapy, which carries the risk of suboptimal outcomes [1–3]. Several scientific societies have suggested a definition of FN based on fever and neutrophil count [4–7]. The international guidelines proposed by these scientific societies recommend the prompt administration of antimicrobials for FN, especially within 60 min in patients with severe sepsis [8, 9]. If the initiation of antimicrobials is delayed, the chances of mortality of patients with FN increase [10, 11]. Therefore, an early diagnosis should be performed to prevent the progression of FN [12].

Various corticosteroids are used in cancer chemotherapy as antiemetic and anticancer drugs and to treat complications. The anti-inflammatory effect of corticosteroids induces suppression of fever [13–15]. The National Comprehensive Cancer Network (NCCN) guideline [4] mentions that the anti-inflammatory effect of corticosteroids could blunt fever responses and any local signs of infection. However, whether corticosteroids influence the onset of FN remains to be studied.

The biological t_1/2_ values of corticosteroids, dexamethasone, prednisolone, and methylprednisolone are in the range of 12–54 h [16]. Because the nadir for neutrophil counts is typically reached 10 to 14 days after the initiation of chemotherapy [17], the fever-suppressive effect of corticosteroid administered within 7 days after the initiation of chemotherapy might not continue until the nadir periods. The aim of this double-center retrospective study was to evaluate whether multiday corticosteroid use in cancer chemotherapy delays the diagnosis of and antimicrobial administration for FN compared with that by corticosteroid use within 7 days after the initiation of chemotherapy.

## Methods

### Definitions

FN was defined as an increase in body temperature to ≥37.5 °C while having a neutrophil count of < 500/μL or < 1000/μL and a predicted decline to ≤500/μL over 48 h according to the Japan Society of Medical Oncology Guideline [7].

In this study, the axillary temperature, routinely measured three times a day in Japan, was selected to evaluate body temperature. It is established that humans have a circadian rhythms for body temperature, and women in the luteal phase have a higher body temperature for a few days [18, 19]. To avoid misinterpreting these influences with increase in body temperature due to infection, the baseline temperature was defined as the highest body temperature during the 7 days before the initiation of chemotherapy.

**Fig. 1:**
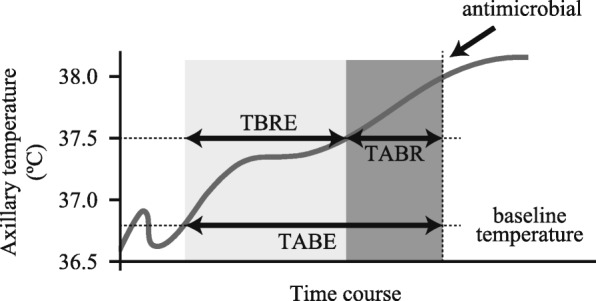
Definition expressing the degree to which febrile neutropenia is blunted. The gray line shows examples of body temperature variation. TBRE: the time to body temperature reaching 37.5 °C from the time when body temperature exceeded the baseline temperature, TABE: the time to antimicrobial administration from the time when body temperature exceeded the baseline temperature, TABR: the time to antimicrobial administration from the time when body temperature reached 37.5 °C. The judgment of exceeding baseline temperature was defined as directly related to reaching 37.5 °C. TABR values can be negative

To evaluate the time of diagnosis and antimicrobial administration, we defined the following three variables (Fig. 1): (1) the time to body temperature reaching 37.5 °C from the time when body temperature exceeded the baseline temperature (TBRE): (2) the time to antimicrobial administration from the time when body temperature exceeded the baseline temperature (TABE): and, (3) the time to antimicrobial administration from the time when body temperature reached 37.5 °C (TABR). To evaluate the time of diagnosis, we surrogated the time to body temperature reaching 37.5 °C which was defined as the diagnosis criteria of FN [7]. TBRE showed whether corticosteroid delays the diagnosis of FN, TABE showed whether corticosteroid delays the antimicrobial administration for FN, and TABR showed whether antimicrobial was administrated immediately after diagnosis.

We evaluated whether the concomitant drugs inhibit cytochrome P450 (CYP) 3A4 by using package inserts of prescription drugs. We investigated the Multinational Association for Supportive Care in Cancer (MASCC) score to predict the grade of FN [20].

To clearly distinguish the effect of the presence/absence of corticosteroids at the nadir periods, we divided the patients into multiday and non-multiday groups based on the duration of corticosteroids use. The multiday group included patients who were administered corticosteroid every day from the initiation of chemotherapy to onset of FN irrespective of its dosage. The non-multiday group included patients who were administered corticosteroid without every day during within 7 days after the initiation of chemotherapy.

### Patients

Patients who were first diagnosed with FN between April 2012 and March 2017 at Kanazawa University Hospital and Kanazawa Municipal Hospital were registered in this study. We excluded patients who were not administered corticosteroids, who were aged less than 18 years, and who had a baseline body temperature of ≥37.5 °C and baseline neutrophil count of < 1500/μL. We also excluded patients who underwent transplantation and radiation therapy, received anticancer drugs after 8 days from the initiation of chemotherapy, immunosuppressive drugs, non-steroidal anti-inflammatory drugs (NSAIDs) including acetaminophen, and granulocyte-colony-stimulating factor (G-CSF), which influenced body temperature and neutrophil count.

### Study design

We conducted a retrospective study by using patients’ computerized medical records. The collected data were age, sex, Eastern Cooperative Oncology Group performance status (ECOG PS), TNM classification of cancer, history of corticosteroid use, type of cancer, chemotherapy regimen, concomitant drugs, body temperature, creatinine clearance (CCr), and total bilirubin (T-Bil). All data was selected from only the first cycle of FN onset for each patient, and referenced the most recent values before the initiation of chemotherapy.

The primary endpoint was determined based on whether multiday corticosteroid use extended the TBRE, TABE and TABR. The secondary endpoint was to identify the risk factors associated with delayed antimicrobial administration.

### Statistical analysis

Patient characteristics were analyzed using Fisher’s exact test and chi-squared test. The relationship between corticosteroid use and TBRE, TABE, and TABR was assessed using the Mann-Whitney *U* test and Kruskal-Wallis test. Correlation between TABE and daily dose of prednisolone in the multiday group was evaluated using Spearman’s rank correlation coefficient. To identify risk factors associated with delayed antimicrobial administration, a multiple logistic regression analysis was performed. Factors for which *P* < 0.300 in the univariate analysis were selected for the multiple logistic regression analysis. Data were analyzed using IBM SPSS Version 24.0 (SPSS Co., Ltd., Tokyo). All statistical difference was assessed by two-side test, and *P* values of < 0.050 were considered statistically significant.

### Ethics statement

The protocol was approved by the ethics committee of Kanazawa University (approval no. 2017–040) and the ethics committee of Kanazawa Municipal Hospital (approval no. 427–12-1). All work was conducted in accordance with the Declaration of Helsinki and ethical principles for clinical research.

## Results

### Patients

**Fig. 2:**
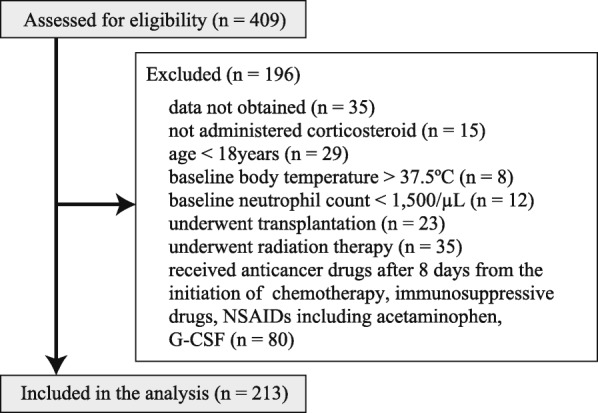
Flow diagram showing patient selection. The number of patients who were enrolled and analyzed in the study is described. The number of excluded patients and reasons for exclusion are also described. The exclusion criteria were duplicates. NSAID: non-steroidal anti-inflammatory drug, G-CSF: granulocyte-colony-stimulating factor

In total, 409 patients were included in the study. One hundred ninety-six patients were excluded, and 213 patients were included in this analysis (Fig. 2). Patient characteristics are listed in Table 1, and each variable was based on risk factors mentioned in the guideline [4]. The patients were divided into two groups based on the duration of corticosteroid, i.e., whether it was multiday or not. All patients in the multiday group were administered prednisolone once or twice a day. Patients in the non-multiday group were administered corticosteroids within 7 days after the initiation of chemotherapy. In the multiday group, 11 patients were complicated with interstitial pneumonia and seven patients received a docetaxel and prednisolone regimen for prostate cancer. All these 18 patients were male. Therefore, a significant difference between the two groups was only in terms of sex (*P* = 0.014). The number of male was 28/41 (68%) in the multiday group and 79/172 (46%) in the non-multiday group.

**Table 1:** Patient characteristics

No. of patients (%)^a^			
Variable	Multiday^b^	Non-multiday^c^	*P*
	(*n* = 41)	(*n* = 172)	
Age (year)			
Median (range)	65(42–87)	62(24–85)	
< 65	20(49)	99(58)	0.38^d^
Sex			
Male	28(68)	79(46)	0.014^d^
ECOG PS			
0	15(37)	66(38)	0.52^e^
1	18(44)	83(48)	
2	7(17)	16(9.3)	
3	1(2.4)	7(4.1)	
CCr (mL/min)^f^			
Median (range)	71.2(39.5–98.0)	74.9(31.8–99.8)	
< 50	3(7.3)	18(11)	0.77^d^
T-Bil (mg/dL)			
Median (range)	1.1(0.2–2.1)	0.9(0.1–2.4)	
< 2	39(95)	162(94)	1.0^d^
FN rate of regimen^g^			
Low (< 10%)	25(61)	104(61)	0.92^e^
Moderate (10 to < 20%)	13(32)	52(30)	
High (≥20%)	3(7.3)	16(9.3)	
Stage			
II	8(20)	25(15)	0.072^e^
III	9(22)	71(41)	
IV	24(59)	76(44)	
CYP3A4 inhibitor			
Use	6(15)	22(13)	0.80^d^
MASCC score			
High risk (≤20)	16(39)	77(45)	0.60^d^
Blood culture			
Positive	6(15)	13(7.6)	0.21^d^

^a^The sum of the percentages may not equal 100% because of rounding off

^b^The multiday group included patients who were administered corticosteroid every day from the initiation of chemotherapy to onset of FN irrespective of its dosage

^c^The non-multiday group included patients who were administered corticosteroid without every day from the initiation of chemotherapy to onset of FN irrespective of its dosage

^d^Fisher’s exact test

^e^chi-squared test

^f^The values were calculated using the Cockcroft-Gault formula

^g^Each rate was based on previous clinical studies [2–6]

*ECOG PS* Eastern Cooperative Oncology Group performance status, *CCr* creatinine clearance, *T-Bil* total bilirubin, *FN* febrile neutropenia, *CYP* cytochrome P450, *MASCC* Multinational Association for Supportive Care in Cancer

### Relation between corticosteroid use and TBRE, TABE, and TABR

TBRE, TABE, and TABR were evaluated in both the multiday and non-multiday groups (Table 2). In the multiday group, TBRE and TABE were significantly extended compared with those in the non-multiday group, with 0.64 and 0.60 days (*P* = 0.002 and *P* < 0.001), respectively. Intergroup differences in terms of TABR were not significant (Table 2). The details of corticosteroid use without those of the multiday group are summarized in Table 3. There were four categories of corticosteroid use: day 1, days 1–3, days 1–5, and days 1–7. Intergroup differences in terms of TBRE, TABE, and TABR on day 1, days 1–3, days 1–5, and days 1–7 were not significant.

**Table 2:** Variation in body temperature and time to antimicrobial administration

Duration of corticosteroid use	TBRE	TABE	TABR
	day	day	day
Multiday^a^ (n = 41)	1.51(0–3.67)	1.70(0–4.11)	0.30(− 0.59–0.91)
Non-multiday^b^ (n = 172)	0.87(0–3.43)	1.10(0–3.97)	0.22(− 0.66–0.89)
*P* ^c^	0.002	< 0.001	0.41

Values are median (range)

^a^ The multiday group included patients who were administered corticosteroid every day from the initiation of chemotherapy to onset of FN irrespective of its dosage

^b^ The non-multiday group included patients who were administered corticosteroid without every day from the initiation of chemotherapy to onset of FN irrespective of its dosage

^c^ Mann-Whitney *U* test

TBRE: the time to body temperature reaching 37.5 °C from the time when body temperature exceeded the baseline temperature

TABE: the time to antimicrobial administration from the time when body temperature exceeded the baseline temperature

TABR: the time to antimicrobial administration from the time when body temperature reached 37.5 °C

Baseline temperature: the highest body temperature during 7 days before the initiation of chemotherapy in each patient

**Table 3:** Variation in body temperature and time to antimicrobial administration in detail without the multiday group

Duration of corticosteroid use	TBRE	TABE	TABR
	day	day	day
day 1 (*n* = 108)	0.80(0–3.34)	1.05(0–3.66)	0.23(− 0.66–0.83)
days 1–3 (*n* = 12)	0.87(0.12–1.57)	1.16(0.09–1.78)	0.22(− 0.21–0.56)
days 1–5 (*n* = 45)	0.89(0–3.43)	1.10(0–0.77)	0.20(− 0.50–0.77)
days 1–7 (*n* = 7)	1.43(0.08–3.08)	1.38(0.51–3.97)	0.10(− 0.16–0.89)
*P* ^a^	0.52	0.71	0.93

Values were median (range)

a Kruskal-Wallis test

TBRE: the time to body temperature reaching 37.5 °C from the time when body temperature exceeded the baseline temperature

TABE: the time to antimicrobial administration from the time when body temperature exceeded the baseline temperature

TABR: the time to antimicrobial administration from the time when body temperature reached 37.5 °C

Baseline temperature: the highest body temperature during 7 days before the initiation of chemotherapy in each patient

### Correlation between TABE and daily dose of prednisolone in the multiday group

**Fig. 3:**
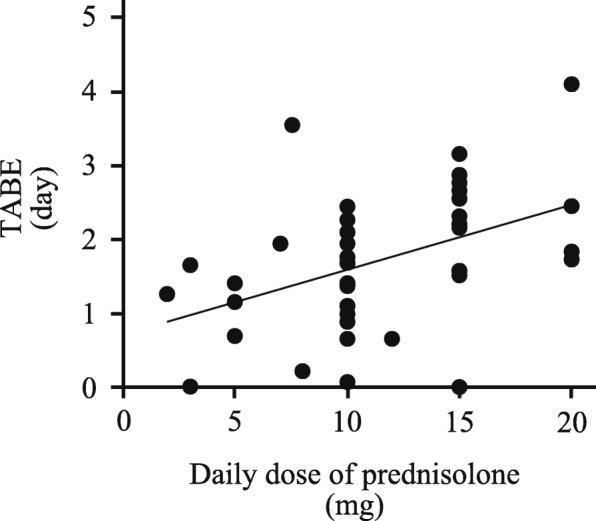
Correlation between TABE and daily dose of prednisolone in the multiday group. TABE increased with an increase in the daily dose of prednisolone (*R* = 0.45, *P* = 0.003, Spearman’s rank correlation coefficient). TABE: the time to antimicrobial administration from the time when body temperature exceeded the baseline temperature

In the multiday group, all patients were administered prednisolone and the dose range was 2 to 20 mg/day. Figure 3 indicates that TABE significantly increased with an increase in the daily dose of prednisolone (*P* = 0.003, *R* = 0.45).

### Univariate and multivariate analyses of risk factors for delayed antimicrobial administration

In the univariate and multivariate analyses, we divided patients into “fast” and “late” groups based on the median TABE, 1.20 days. In the univariate analysis, the factors with *P* values < 0.300 were sex, duration of corticosteroid use, CYP3A4 inhibitor use, and MASCC score (Table 4). These factors were included in the multivariate analysis (Table 4), and the results indicated that the duration of corticosteroid use was an independent risk factor for delayed antimicrobial administration (odds ratio = 3.94; 95% confidence interval = 1.80–8.62; *P* < 0.001).

**Table 4:** Univarate and multivariate analyses of risk factors for delaying antimicrobial administration

Variable	Univariate analysis		*P*	Multivariate analysis	
	No. of patients (%)^a^			OR (95% CI)	*P* ^e^
	fast^b^	late^b^			
	(*n* = 107)	(*n* = 106)			
Age (year)					
< 65	61(57)	58(55)	0.78^c^		
Sex					
Male	58(54)	49(46)	0.27^c^	0.71(0.50–1.20)	0.16
ECOG PS					
0	41(38)	40(38)	0.77^d^		
1	48(45)	53(50)			
2	13(12)	10(9.4)			
3	5(4.7)	3(2.8)			
CCr (mL/min)^f^					
< 50	9(8.4)	12(11)	0.50^c^		
T-Bil (mg/dL)					
< 2	5(4.7)	7(6.6)	0.57^c^		
FN rate of regimen^g^					
Low (< 10%)	64(60)	65(61)	0.48^d^		
Moderate (10 to < 20%)	31(29)	34(32)			
High (≥20%)	12(11)	7(6.6)			
Stage					
II	18(17)	15(14)	0.79^d^		
III	41(38)	39(37)			
IV	48(45)	52(49)			
Duration of corticosteroid use					
Multiday^h^	11(10)	30(28)	0.001^c^	3.94(1.80–8.62)	< 0.001
CYP3A4 inhibitor					
Use	11(10)	17(16)	0.23^c^	2.04(0.86–4.84)	0.11
MASCC score					
High risk (≤20)	52(49)	41(39)	0.17^c^	1.38(0.78–2.46)	0.27
Blood culture					
Positive	10(9.3)	9(8.5)	1.0^c^		

^a^ The sum of the percentages may not equal 100% because of rounding off

^b^ The classification into “fast” and “late” groups was based on the median TABE, 1.20 days

^c^ Fisher’s exact test

^d^ chi-squared test

^e^ Logistic regression analysis

^f^ The values were calculated using the Cockcroft-Gault formula

^g^ Each rate was based on previous clinical studies [2–6]

^h^ The multiday group included patients who were administered corticosteroid every day from the initiation of chemotherapy to onset of FN irrespective of its dosage

*OR* odds ratio, *CI* confidence interval, *ECOG PS* Eastern Cooperative Oncology Group performance status, *CCr* creatinine clearance, *T-Bil* total bilirubin, *FN* febrile neutropenia, *CYP* cytochrome P450, *MASCC* Multinational Association for Supportive Care in Cancer

TABE: the time to antimicrobial administration from the time when body temperature exceeded the baseline temperature

## Discussion

In this analysis, we found that multiday corticosteroid use significantly prolonged TBRE and TABE. Thus, multiday corticosteroid use was selected as a risk factor of prolonged TABE in multivariable analysis.

Multiday corticosteroid use, in which prednisolone was administered once or twice a day to all patients, significantly prolonged TBRE and TABE in comparison with non-multiday corticosteroid use. Since the biological t_1/2_ of prednisolone is 12–26 h [16], the fever-suppressive effect lasts for more than half a day [14]. In this study, the results for TBRE were along expected lines. Because there was no significant difference in TABR, physicians administered antimicrobials based on only body temperature > 37.5 °C. The current guidelines recommend that judgment of antimicrobial administration for patients receiving corticosteroids should be based on not only the body temperature variation but also the neutrophil count and the general clinical course [4]. The results of this study primarily support this recommendation. On the other hand, the influence of corticosteroids’ immunosuppressive effects on body temperature variation should be considered. At the lowest neutrophil counts, the course of fever in the multiday group was influenced by the immunosuppressive effects of corticosteroids, leading to an earlier onset of infection-related symptoms in comparison with that in the non-multiday group. As a result, the TBRE in the multiday group was expected to be shorter than that in the non-multiday group, which was not affected by the immunosuppressive effect of corticosteroids. However, the TBRE in the multiday group was significantly longer than that in the non-multiday group in this study (Table 2). The anti-inflammatory effect of corticosteroids could blunt fever response and any localizing signs of infection [4]. These results suggest that the use of corticosteroids in the multiday group blunted a fever induced by some infection in FN patients. Furthermore Fig. 3 indicates that the degree of fever suppression depends on the dose of the corticosteroid. This finding is a very important point for management of FN in patients with concurrent administration of chemotherapy and daily corticosteroid administration. Medical staff should always keep the duration and dosage of corticosteroids in mind.

Only multiday corticosteroid use was a significant risk factor for prolonged TABE in multivariable analysis. Unexpectedly, CYP3A4 inhibitor use was not an independent risk factor for prolonged TABE, even though CYP3A4 inhibitors show the ability to increase the blood concentration of corticosteroids. For example, itraconazole and ketoconazole increased the total area under the plasma methylprednisolone concentration-time curve 3.9-fold and 1.4-fold, respectively, in comparison with the placebo [21, 22]. However, detail such as the dosage of CYP3A4 inhibitors was not collected. Although, the degree of interaction could be relatively small to elevate the blood concentration of corticosteroids in this study, it is necessary to clarify these influences in a future study.

Infections in neutropenic patients can progress rapidly, leading to hypotension and other life-threatening complications. Early detection and treatment, which involves prompt initiation of empirical systemic antibacterial therapy, of neutropenic fever is critical in order to avoid progression to a sepsis syndrome and possibly death [4, 12]. In the presence of septic shock, each hour’s delay in initiating administration of effective antimicrobials is associated with a measurable increase in mortality [9, 23, 24]. In this study, we first noted a difference of 0.60 days in the TABE between the multiday and non-multiday groups. This indicated that the mortality of patients receiving concomitant corticosteroid regimen could increase. Therefore, more attention should be paid to the concomitant drugs, especially corticosteroids, when chemotherapy is performed.

Several limitations of this study should be acknowledged. First, we used three new definitions, TBRE, TABE, and TABR. Since body temperature was measured three times a day, the time beyond the baseline temperature and reaching 37.5 °C could not be precisely determined. Furthermore, frequent thermometry was provided to patient who presented the clinical symptom of severe infection and therefore the medical staff might discover body temperature reaching 37.5 °C at an early stage. The validity of TBRE, TABE, and TABR has not been fully confirmed, because we defined that criterion for this study. It is thus important to further discuss in future studies. Second, since more than 95% of patients who were underwent cancer chemotherapy and showed FN received corticosteroids, we could not consider the patients not taking corticosteroids as a control group. As shown in Table 3, TBRE and TABE tended to be prolonged in the days 1–7 group among the non-multiday groups. It is possible that medical staff should note about the patients administered corticosteroid at near the nadir of neutrophil. Finally, this study was retrospective in nature. The causative pathogens and infection sources of FN were not completely clarified, and minor differences may have been present in patient characteristics. Further studies are required to identify the timing of the most suitable antimicrobial administration to patients receiving multiday corticosteroids.

## Conclusion

The findings of this study indicate that multiday corticosteroid use in cancer chemotherapy delays the diagnosis of and antimicrobial administration for FN. Moreover, multiday corticosteroid use is the only risk factor for delayed antimicrobial administration. Although several guidelines recommend that judgment of antimicrobial administration for patients receiving corticosteroids should be based not only on the body temperature variation but also the general clinical course, there has been no evidence for this approach. This study is the first to show evidence in support of this recommendation.

## Abbreviations

CCr: Creatinine clearance
CYP: Cytochrome P450
ECOG PS: Eastern Cooperative Oncology Group performance status
FN: Febrile neutropenia
G-CSF: Granulocyte–colony-stimulating factor
MASCC: Multinational Association for Supportive Care in Cancer
NCCN: The National Comprehensive Cancer Network
NSAIDs: Non-steroidal anti-inflammatory drugs
TABE: The time to antimicrobial administration from the time when body temperature exceeded the baseline temperature
TABR: The time to antimicrobial administration from the time when a body temperature of 37.5 °C was reached
T-Bil: Total bilirubin
TBRE: The time to reach a body temperature of 37.5 °C from the time when body temperature exceeded the baseline temperature
